# Accuracy of paper-and-pencil systematic observation versus computer-aided systems

**DOI:** 10.3758/s13428-022-01861-0

**Published:** 2022-04-27

**Authors:** Javier Virues-Ortega, Candida Delgado Casas, Neil Martin, Aida Tarifa-Rodriguez, Antonio Jesús Reina Hidalgo, Alison D. Cox, José I. Navarro Guzmán

**Affiliations:** 1 Universidad Autónoma de Madrid, Madrid, Spain; 2 University of Auckland, Auckland, New Zealand; 3 Universidad de Cádiz, Cádiz, Spain; 4 Behavior Analyst Certification Board, Littleton, Colorado USA; 5 Universidad Nacional de Educación a Distancia, Madrid, Spain; 6 Brock University, St. Catharines, Ontario Canada

**Keywords:** Accuracy, Behavior observation, Computer-aided observation

## Abstract

Computer-aided behavior observation is gradually supplanting paper-and-pencil approaches to behavior observation, but there is a dearth of evidence on the relative accuracy of paper-and-pencil versus computer-aided behavior observation formats in the literature. The current study evaluated the accuracy resulting from paper-and-pencil observation and from two computer-aided behavior observation methods: The Observer XT® desktop software and the Big Eye Observer® smartphone application. Twelve postgraduate students without behavior observation experience underwent a behavior observation training protocol. As part of a multi-element design, participants recorded 60 real clinical sessions randomly assigned to one of the three observation methods. All three methods produced high levels of accuracy (paper-and-pencil, .88 ± .01; The Observer XT, .84 ± .01; Big Eye Observer, .84 ± .01). A mixed linear model analysis indicated that paper-and-pencil observation produced marginally superior accuracy values, whereas the accuracy produced by The Observer XT and Big Eye Observer did not differ. The analysis suggests that accuracy of recording was mediated by the number of recordable events in the observation videos. The implications of these findings for research and practice are discussed.

**Supplementary Information:**

The online version contains supplementary material available at 10.3758/s13428-022-01861-0.

Focus on direct observation of behavior is a key method for experimental psychology, clinical psychology, education, comparative psychology, ethology, behavior analysis, and numerous other fields (Behavior Analyst Certification Board, 2017; Haynes & O’Brien, 2000; Hintze, 2004; Yasukawa & Bonnie, 2017). Examples of the range of behaviors that are commonly observed by researchers include classroom teaching (Gargani & Strong, 2014), parent-child interactions (Hudson & Rapee, 2001), patient-doctor interactions (Hayward et al., 2015), and animals across a wide range of settings and species (e.g., Bailey et al., 2004). Systematic behavior observation is paramount to the assessment and treatment of clinically important behavior such as sleep problems (Roth et al., 1976), feeding disorders (Piazza, 2008), and problem behavior of children, adolescents, and adults (Hanley et al., 2013). Access to mobile and computer-based behavior observation applications has made the use of such technology near-universal, yet research on its ability to enhance the accuracy of behavior observation has remained rare.

Numerous software-based applications for animal and human behavior observation have become available over the last few decades: Big Eye Observer® (ABA España, 2019), CATOS (Oh & Fitch, 2017), Countee (Gavran & Hernandez, 2020), EthoVision XT® (Noldus Information Technology, 2021), JWatcher (Blumstein et al., 2006), ObsWin (Martin et al., 1999), Solomon Coder (Péter, 2019), The Observer XT® (Noldus Information Technology, 2019), ZoneMinder (Farrimond et al., 2009), to mention just a few examples (also see early applications in Bass, 1987, and Kahng & Iwata, 1998). In the early 2000s, studies began to report the use of desktop and handheld computer applications for conducting systematic observation. Jackson and Dixon (2007) used an application created with Microsoft Visual Basic for recording data during direct observation of functional analysis sessions using a handheld computer. The software produced frequency- and interval-based observations with output files that could be imported into statistical and graphing software packages. Crowley-Koch and Van Houten (2013) described a variety of technological solutions aimed at facilitating the collection of data via direct observation, including video-synced software applications and internet-based applications. Software, such as The Observer XT (Noldus Information Technology, 2019), has been used widely in studies involving systematic observation in the areas of animal learning and experimental psychology (e.g., Franchi et al., 2016), organizational and consumer behavior (e.g., Allen et al., 2015), clinical interaction in psychotherapy (e.g., Pardo-Cebrian et al., 2021; Virues-Ortega et al., 2011), intervention studies in children and adults with and without developmental and intellectual disability (Hutman et al., 2012; Meirsschaut et al., 2011; Mossman, 2011; Naber et al., 2008), and studies in dementia (Moyle et al., 2014), among numerous other applications. These studies show the range of applications of such systems, and they have all consistently reported high levels of interobserver agreement for computer-aided observations.

Several authors have noted the advantages of computer-aided observation over more traditional methods, highlighting the convenience of electronic data storage and analysis, usability of computer interfaces, and discreteness of handheld devices (e.g., Tarbox et al., 2010). In spite of the apparent advantages of computer-aided data collection systems, very little research has empirically evaluated their impact on the accuracy of the data collection process (Jackson & Dixon, 2007; Kahng & Iwata, 1998; Tarbox et al., 2010).

Wessel (2015) has described the advantages of computer-assisted direct-observation apps, relative to desktop computer-based systems, which may allow for accurate real-time coding of events in vivo. Real-time observation apps are highly accessible via smartphones and simplify the ethical concerns derived from video-based observation. They also provide the observer with the opportunity to record contextual information that could be easily missed in video-based observations. A key consideration when comparing in vivo observation with recorded retrospective video-based coding is the ability with the latter to use video playback functions (i.e., pausing, rewinding, use of slow motion, advancing frame-by-frame), as these may have the potential to optimize accuracy, particularly during eventful sessions. Yet, the potential impact of session *busyness* on accuracy in terms of in vivo versus video playback observation modes has not been evaluated in the literature.

Very few studies have compared the differences between paper-and-pencil and computer-aided systematic observation. In a notable exception, Tarbox et al. (2010) compared these in the context of services being provided to children diagnosed with autism spectrum disorder. A total of four participants used either paper-and-pencil or a handheld computer to observe across a range of sessions. Interestingly, the results suggested that computer-aided data collection was less time-efficient than paper-and-pencil methods. The accuracy of paper-and-pencil and computer-aided observation ranged from 98 to 100% and from 84 to 95% respectively, suggesting slight superiority of paper-and-pencil observation. The authors suggested that in this particular arrangement observers required less time to record their observation with the paper-and-pencil format, whereas the computer-aided format required the observer to sequentially select multiple keys and screens. While data collection with the handheld computer application was somewhat slower, it did allow for automated graphing. The findings from Tarbox et al. (2010) require replication with alternative computer-aided observation systems, including those with simplified interfaces. In addition, participants in Tarbox et al. (2010) recorded live sessions. Therefore, it was not possible to discard other factors that may have had an effect on the accuracy of recording such as the number of events recorded and the emission rates of those events.

Given the scarcity of studies devoted to the evaluation of data collection systems for behavior observation, a systematic comparison of some of the different formats of observation would be highly informative. In the current study we evaluated the relative accuracy of using the paper-and-pencil method, desktop computer software that allowed retrospective video analysis (The Observer XT), and a mobile app (Big Eye Observer). The current selection of systems was intended to capture the range of platforms and capabilities of existing systems (see Table A in the Supplementary Online Material for a comparison of representative systems). The study adds to the literature by using a comparable set of observation sessions across participants, controlling for the participants’ behavior observation training and experience, using a larger sample size than has been reported in previous studies, and accounting for the number of recordable events in a session and the observers’ use of video playback functions.

## Material and methods

### Participants and setting

Twelve female students (mean age 23.2 years, range 20–41) without past experience in behavior observation and enrolled in a postgraduate course in applied psychology in New Zealand participated in the study. Students enrolled in the course (*n* = 16) received an email inviting them to participate (recruitment period, April 1, 2019, through April 30, 2019). Observation sessions took place in a quiet laboratory space on university premises and at the participants’ homes. To prevent observer bias, participants were blind to the goals of the study and did not receive performance-related feedback over the course of the study (Lerman et al., 2010). One individual declined to continue to participate shortly after the study started (P7). One additional participant did not follow the expected order of sessions resulting in their data being excluded from further analysis (P10). We conducted Monte Carlo simulations to study a priori the power of the intended mixed model analysis and sample size (Gelman & Hill, 2008). The power achieved was above .90 for a sample size of both 10 and 15 subjects, assuming a .05 mean difference in accuracy across the levels of the main fixed-effect factor (observation method) and 60 successive participant-nested measurements. Sample size was established a priori and was not subsequently modified.

The study protocol was approved by the social sciences ethics committee of the University of Manitoba (Canada), the human ethics committee of The University of Auckland (New Zealand), and the ethics committee of the University of Cadiz (Spain). All participants signed an informed consent form. The current report adheres to the TREND statement (Jarlais et al., 2004). Dataset files are available from Virues-Ortega et al. (2022b).

### Materials

#### Observation videos

Participants observed 60 distinct 5-min videos over the course of the study. All videos had a resolution of 720 × 480 pixels and a frame rate of 25 frames per second. The video collection was originally obtained as part of the study by Cox and Virues-Ortega (2021). The videos portrayed actual *demand* sessions from experimental functional analyses and featured a range of problem behaviors and clients. The functional analysis sessions presented in the videos followed the procedures described by Iwata et al., 1994) with the procedural adaptations specified by Cox and Virues-Ortega (2021). Demand sessions were chosen because there were a reasonably high number of recordable events in all sessions—observation sessions with a relatively low number of events can produce artificially high levels of agreement/accuracy when using the block-by-block method of agreement (cf. Mudford et al., 2009; Page & Iwata, 1986) that was used in this study. Target events included *compliance*, *praise*, and *demand*, and each video featured one of a number of distinct problem behavior topographies (e.g., self-injurious, destructive, aggressive behaviors). Definitions of all target events, consistent with the taxonomy by Ray et al. (2011), are available on request from the corresponding author. The mean number of recordable events across each 5-min video was 62.5 (range 20–134). Data from each of the participants based on the observation of four specific videos were excluded from the analysis after the study was completed—the camera angle of these particular videos made some of the target events visually ambiguous.

#### Paper-and-pencil observation

Participants used observation data sheets with 30 separate rows for each of the 10-s intervals required for recording a 5-min video. Each datasheet had columns indicating the interval number, interval start and end, and separate columns for each target event. Participants used VLC Media Player, version 3.0.6 (VideoLAN Organization, 2019) to play the videos to be recorded using paper-and-pencil. The VLC Media Player was chosen because time elapsed and time remaining counters are clearly visible during playback and also to standardize the way in which the videos were displayed. Additionally, the media player had the specific video playback functions that could be used.

#### The observer XT

Participants used The Observer XT version 14.2 observation software. The observation module displays the session’s video, a video timeline, keys for each observation code (i.e., target events), and video playback functions. The research team provided participants with instructions to create an event observation coding scheme that would include all relevant target events to be recorded as discrete events (*point events*). The research team subsequently verified that the observation coding schemes were set up correctly. Output files in text format were produced for further analysis.

#### Big eye observer

The Big Eye Observer iPhone**®**/iPad**®** application is a single-screen systematic observation app that allows the recording of up to 12 distinct events concurrently using a variety of methods (i.e., frequency/count, partial interval, total interval, duration). The research team provided participants with instructions to create an event observation template that would include all relevant target events. The research team subsequently verified that the observation templates were set up correctly. All target events were recorded as discrete events (*frequency events*). Output files were subsequently emailed in text format for further analysis.

### Criterion reference for videos

All videos were observed and data collected on the four target events in each video independently by two trained observers with over 10 years of experience in systematic observation. Observers used a Microsoft Excel spreadsheet to input their observations. After completing the observations, the observers checked their agreement in each of the videos on an interval-by-interval basis with the videos divided up into 10-s intervals. Any disagreement on the number of events for each of the four target events was discussed and consensus reached. Most disagreements had to do with events occurring around the start or end of a 10-s interval, events occurring in quick succession, or events that were difficult to discriminate (visually or audibly) and which often required frame-by-frame replay until consensus could be reached. The resulting *criterion reference* recordings provided the agreed number of recordable events for each interval within each video and were used to assess the accuracy of the data collected by participants from all observation sessions using one of the three different observational recording methods (paper-and-pencil, The Observer XT, Big Eye Observer).

### Accuracy

Accuracy was calculated using the block-by-block method of analysis. The 5-min videos were divided into 30 10-s intervals, and each 10-s interval was scored by dividing the smaller number of events recorded in each interval by the larger number of events, or scored as 1 if the number of events were identical for the participant and reference recording. This process was repeated for each of the four target events recorded. An accuracy index value for each session was then calculated by summing the scores for each of the 30 intervals for each of the four target events and dividing by 120 (the total number of assessed intervals).

### Design

Participants conducted 20 observation sessions for each of the observation methods (paper-and-pencil, The Observer XT, Big Eye Observer) totaling 60 sessions, and these were randomly alternated as part of a multi-element experimental design embedded in a within-subjects design (Kazdin, 2011). In order to evaluate whether observation methods would yield differential results under (simulated) in vivo conditions or when able to use the video playback functions, we used the ability to use video playback functions as a secondary independent variable. Specifically, participants were told either that they could use video playback functions (*video playback phases*) or to refrain from using the playback functions (*in vivo proxy phases*) across four successive 15-session study phases. Each phase included five sessions for each observation method randomly sequenced within the 15-session phase. Phases were alternated as part of an ABAB reversal design. In order to control for the effects of phase order, participants 1, 3, 5, 9, and 11 (P1, P3, P5, P9, P11) initiated the reversal design with the video playback phase, whereas P2, P4, P6, P8, and P12 started with the in vivo proxy phase.

### Procedure

Participants had no past experience with observation systems. All participants attended a session with the principal investigator where the materials of the observation training protocol by Dempsey et al. (2012) were presented. This protocol contains six 10-min videos showing a range of problem behavior and environmental events (instructions, praise, demand, attention). Each video has an increasing number of events and behavior codes relative to the preceding one. For example, video #1 has a single target behavior and a total of 30 recordable events, whereas video #6 has six distinct target behaviors and a total of 178 recordable events. Participants had to record the target behaviors from each video but were instructed to advance to the next video only if they had reached an accuracy index of .90 or higher for all target behaviors. Lower accuracy would result in the participant repeating the observation and recording for that video. Participants had a mean accuracy of .97 (range .94–.99) in their last attempt in all training videos, required a mean 8.6 sessions to attain the criterion for the six videos (range 6–13), and required a mean training time of 83.8 min (range 60–130). Participants took between 1 and 4 days to complete the training. All data collection occurred using printed observation data sheets.

After completing the behavior observation training protocol, participants received written instructions, a 1-h video tutorial, and a 2-h workshop led by the principal investigator. The written instructions primarily covered the process of accessing the study materials, how to use the relevant observational tools, and the operational definitions of the target events. The video tutorial was on the use of VLC Media Player, The Observer XT, and Big Eye Observer for the purposes of the study. The workshop was intended to provide hands-on training for using the three systems and to troubleshoot any technical or logistical difficulties. Participants were able to contact the research team throughout the study via phone or email to have their questions or concerns addressed.

Participants were allowed to use playback functions (i.e., pausing, rewinding, use of slow motion, advancing frame-by-frame) during video playback phases. This was also true for the Big Eye Observer app, which had an option to “pause recording.” While the app was paused, participants would have been able to use video playback controls in the video player. The only caveat of using this approach with the app was that participants would need to sync the video time counter with that of the app when resuming the observation. The video playback functions were integrated in The Observer XT interphase.

Participants conducting the study at the university had access to a desktop computer with VLC Media Player and The Observer XT installed, and a 9.7-inch iPad with the Big Eye Observer installed. In addition, participants conducting part of the observation at home received the temporary loan of a laptop computer with VLC Media Player and The Observer XT installed, and an activation code for the Big Eye Observer app to run on their iPad or iPhone. Participants who did not own an iPad or iPhone and were conducting any observation from home were loaned a sixth-generation 9.7-inch iPad with the Big Eye Observer installed.

Participants were instructed to use the VLC Media Player during both their paper-and-pencil and Big Eye Observer observation sessions and asked to keep the relative size of the video window on their screen constant when using all three observation methods. Participants were instructed to conduct observations in a quiet environment and to keep a handout with the operational definitions of the target behaviors within their reach for easy reference. The observation data sheets used during paper-and-pencil observation sessions were subsequently scanned and the participants received a personalized secure link to upload the scanned files and The Observer XT and Big Eye Observer output files.

Participants conducted the observation in bouts that typically included 10 to 15 observation videos with minimal breaks in between. Participants engaged in these observation bouts across several days until the study protocol was completed. The mean number of successive calendar days to complete the study protocol was 9.4 (range 1–21).

### Procedural integrity

To evaluate whether study procedures were followed as intended, participants were asked to fill out a form as they progressed through the study noting the sequence, start time, and end time of behavior observation sessions. The percentage of observation sessions conducted in the expected sequence was calculated, along with the percentage of missing sessions and the percentage of duplicate sessions. The percentage of observation sessions completed using the expected sequence was 100% for all participants. The mean percentage of missing sessions across participants was only 2.7% (range 0–16.7%). Only two duplicate sessions were identified in the dataset, both produced by P3, and only the session recorded first was included in the analysis and the duplicate sessions were discarded. Neither an iPad nor iPhone could be secured for P5, and so only paper-and-pencil and Observer XT sessions were completed. P11 completed all sessions but failed to properly upload the data files for 10 of the Observer XT sessions.

**Fig. 1:**
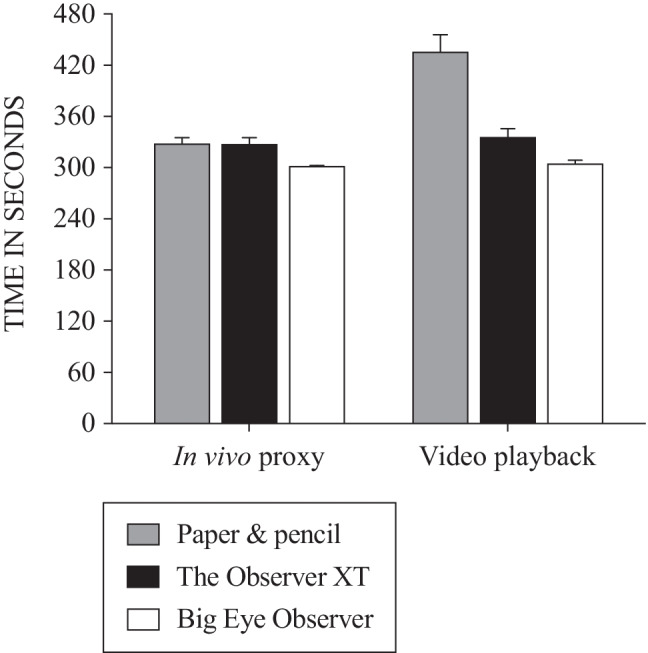
Observation time across phases and observation methods. *Note.* All means and standard errors

Because using the video playback functions during those phases was dependent on the participants following the instructions provided, session start and end times were extracted from The Observer XT and Big Eye Observer data outputs and from the paper-and-pencil data sheets such that the total session time spent recording each video could be calculated. This helped to indirectly verify whether the participants used any playback functions and whether playback functions were only used during the expected phases (i.e., session lengths during the video playback phases should have been consistently longer than the length of the 5-min video). Figure 1 presents the mean observation time for the in vivo proxy and video playback phases across observation methods. The mean observation time during in vivo proxy phases for each observation method suggests that participants did not use the video playback functions while using Big Eye Observer (302.00 ± 0.78 s), but did use them (albeit sparingly) during the paper-and-pencil (328.48 ± 6.52 s) and Observer XT sessions (327.95 ± 7.22 s). Interestingly, a similar pattern was observed in The Observer XT (336.16 ± 9.58 s) and Big Eye Observer sessions (304.91 ± 3.66 s) during the actual video playback phases. The observation time data seems to suggest that participants only used the intended video playback functions comprehensively for the paper-and-pencil sessions (436.04 ± 19.78 s).

### Data processing

The final dataset included approximately 600 datasheets in three different formats. All data files were reviewed manually for any missing or duplicate sessions. All data were either tabulated or imported into Microsoft® Excel spreadsheets. An Excel Visual Basic for Applications script was developed to transform all files into a single format, import all into one spreadsheet, extract the relevant data, compute the number of events for each of the four target events for each 10-s interval for each 5-min session, and conduct the accuracy analyses (i.e., compare the participants’ data with the criterion reference for that session).

### Statistical analysis

The time-series nature of the data, lack of a normal distribution, and the presence of missing values supported using mixed linear models with the current dataset. Participant number was used as the subject variable, session number as the time-based variable, and accuracy as dependent variable. A first-order autoregressive covariance structure rendered the best goodness-of-fit values during the model development process (*ρ* = –0.15 ± 0.07). Observation method (paper-and-pencil, The Observer XT, Big Eye Observer), number of recordable events, video mode experimental phase (video playback, in vivo proxy), and their interaction were added as fixed-effect factors. Maximum likelihood estimation was used to determine the model’s parameters, and pairwise comparisons were computed across observation methods and video mode experimental phase. Pairwise coefficients were expressed using the same metric as the dependent variable (range 0–1). Factors and co-variables failing to improve goodness of fit by a minimum of two units using Akaike’s information criterion (AIC) were excluded from the model (Akaike, 1974; Burnham & Anderson, 2002). This resulted in phase order, reversal design order, and observation time not being included in the final mixed model analysis as either factors or co-variables. The model goodness of fit was further optimized (AIC, −1133 vs. −908) by adding method and video mode experimental phase as participant-nested factors, and recordable events nested by observation video (i.e., video number). All analyses were conducted with IBM® SPSS® Statistics, version 27 (IBM Corporation, 2021). A *p* value of .05 was used throughout with Bonferroni adjustments for multiple comparisons.

## Results

**Fig. 2:**
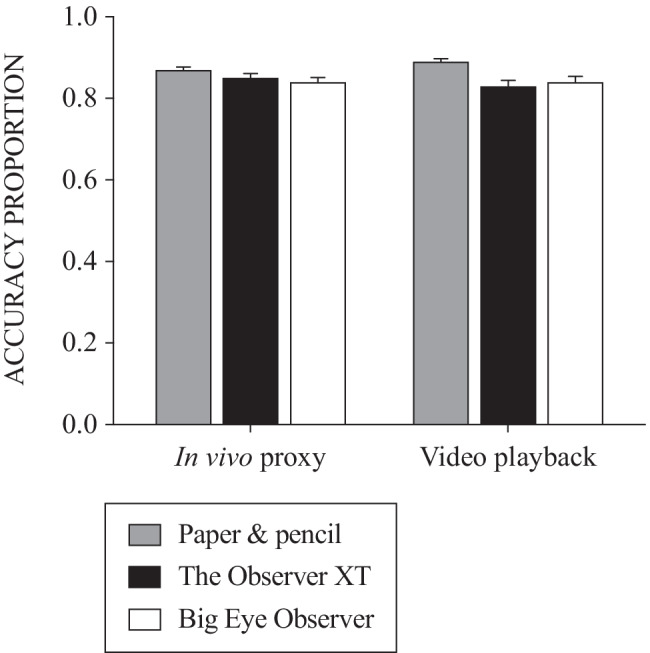
Accuracy across phases and observation methods. *Note.* All means and standard errors

**Fig. 3:**
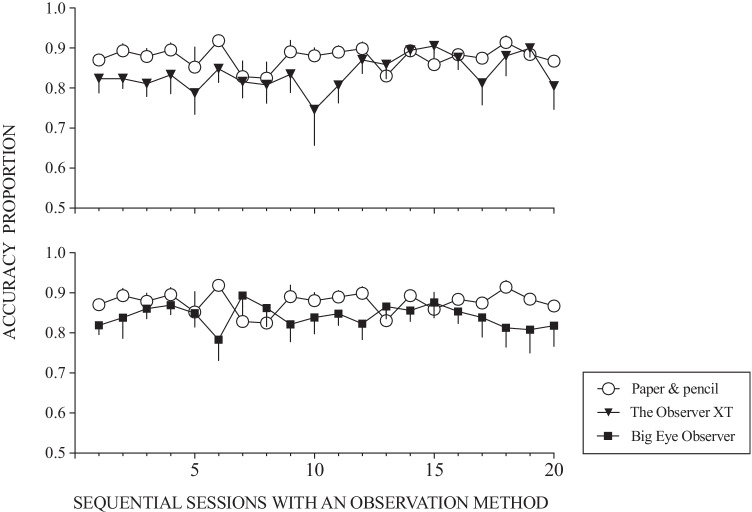
Observation accuracy of The Observer XT® vs. paper-and-pencil (top panel) and of Big Eye Observer® vs. paper-and-pencil (lower panel) across participants over sequential sessions with the observation method. *Notes*. All means and standard errors. The successive order of the multi-element design has been suppressed to allow for data aggregation across participants

Figure 2 shows the mean accuracy data across participants disaggregated by phase and observation method. A preliminary visual analysis of these data suggested relatively high levels of accuracy across all three methods (paper-and-pencil, .88 ± .01; The Observer XT, .84 ± .01; Big Eye Observer, .84 ± .01) although slightly better accuracy for the paper-and-pencil method. This effect was also apparent when the data are aggregated across participants on a session-by-session basis (Fig. 3). However, due to high levels of variability across participants it was not possible to ascertain consistently different levels of accuracy across specific observation methods or experimental phases through the visual analysis of individual participants’ data. Session-by-session graphs for each participant are available on request from the corresponding author.

**Fig. 4:**
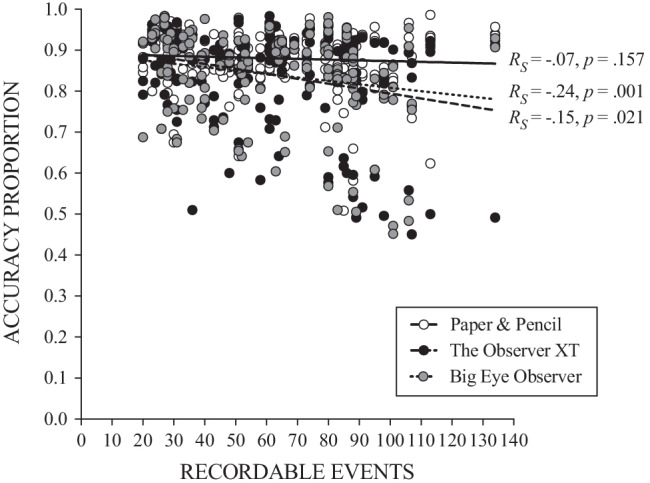
Accuracy by number of recordable events across all observation sessions. *Note*. *R*_S_ = Spearman rank correlation coefficient

The final mixed model analysis (Table 1) confirmed a significant effect of observation method, *F*(2, 543) = 16.022, *p* < .001. Pairwise comparisons indicated that both The Observer XT and Big Eye Observer produced slightly lower levels of accuracy relative to paper-and-pencil observations: paper-and-pencil versus The Observer XT, coefficient = .035 ± .007, *p* < .001; paper-and-pencil versus Big Eye Observer, coefficient = .039 ± .007, *p* < .001. Thus, accuracy decreased by approximately .04 when switching from paper-and-pencil observation to computer-aided observation systems. There were also significant fixed effects for the number of recordable events, *F*(45, 526) = 5.308, *p* < .001, and the interaction of this with observation method, *F*(88, 527) = 1.844, *p* < .001. Figure 4 shows a scatterplot with Spearman rank correlation analysis suggesting that the mediating effect of the number of recordable events was observed primarily in The Observer XT (*R*_S_ = −0.15, *p* = .021) and Big Eye Observer (*R*_S_ = −0.24, *p* = .001) observation methods.

**Table 1:** Linear mixed-effects model for accuracy proportion (*n* = 10)

Fixed effects	*F*	*df*	*p*
Method (A)	16.022	2, 543	< .001
Recordable events (B)	5.308	45, 526	< .001
Video mode (C)	3.016	1, 263	.084
Interaction, A × C	1.844	88, 527	< .001
Interaction, A × C	1.552	2, 537	.213
Fixed effects (nested factors)			
Method (Participant)	11.179	17, 530	< .001
Video mode (Participant)	3.068	9, 256	.002
Recordable events (Video number)	6.467	10, 535	< .001
Pairwise comparisons	Coefficient	*df*	*p*
Video mode			
Video playback vs. in vivo proxy	.011 ± .006	1, 265	.065
Method			
P&P vs. OXT	.035 ± .007	2, 541	< .001
P&P vs. BEO	.039 ± .007	2, 539	< .001
OXT vs. BEO	.003 ± .008	2, 542	1.000

First-order autoregressive covariance structure (*ρ* = −0.15 ± 0.07). The coefficient of pairwise comparisons is the mean difference (first term minus second term) expressed in the metric of the dependent variable. BEO = Big Eye Observer, OXT = The Observer XT, P&P = paper-and-pencil.

Increased observation time in only the paper-and-pencil sessions during the (expected) video playback phases (Fig. 1) is a potential confound to the slightly superior assessed accuracy of paper-and-pencil sessions. However, the video mode experimental phase (video playback and in vivo proxy) was not established as a significant fixed-effect factor in the mixed model analysis, *F*(1, 263) = 3.016, *p* = .084. However, it reached statistical significance when included as a participant-nested factor, *F*(9, 256) = 3.068, *p* = .002, suggesting that video mode may have a mediating role over accuracy for some individuals. The pairwise comparison indicated a trend toward increased accuracy of the video playback mode, coefficient = .011 ± .006, *p* = .065. Finally, adding observation time as a random-effects co-variable had deleterious effects in the model’s fitness.

## Discussion

Assessment, treatment planning, implementation, and evaluation depend heavily on the ability to be able to collect meaningful and robust data through behavior observation, and on the accuracy of those data. Therefore, it is important to maximize the efficiency of behavior observation methods while collecting data reliably and accurately. The rationale for assessing the reliability of data collected in practice is threefold: (1) to check the consistency of observations, (2) to minimize bias, and (3) to verify the adequacy of response definitions (Kazdin, 2011). In the current study, we compared the accuracy of data collected using traditional paper-and-pencil methods with two computer-aided methods. Block-by-block agreement, one method used to assess the *reliability* of behavior observation data, was utilized to assess the *accuracy* of the different systematic observation formats. Specifically, we used the same analysis to compare the data recorded by the participants using different methods against a predetermined criterion reference for each dataset. Our findings suggest that formally trained observers can reach high levels of accuracy with a range of behavior observation methods with minimal accuracy loss that could be attributed to observation difficulty.

**Fig. 5:**
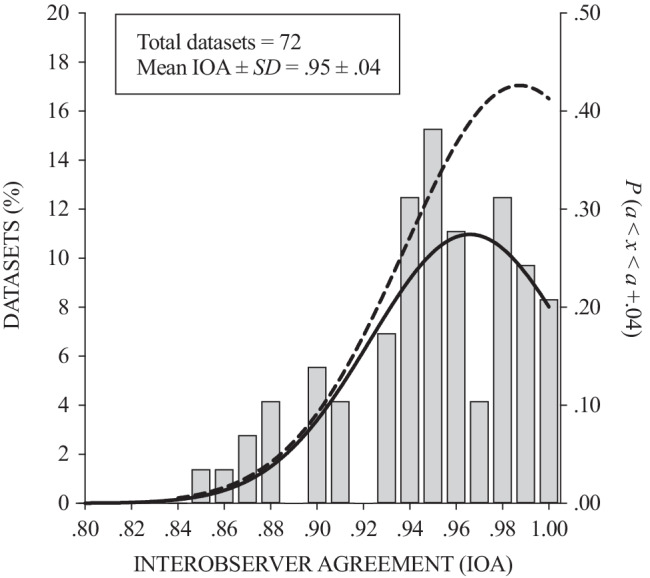
Distribution of interobserver agreement values in the literature. *Notes.* The bars and solid line (left *y* axis) denote the empirical and estimated distribution of interobserver agreement (IOA) values in the literature as reviewed in a selection of the functional analysis literature by Virues-Ortega et al. (2022a). The broken line (right *y* axis) is the cumulative probability function between any IOA value *a* and an IOA value *a* + .04 (according to the probability density function for a truncated normal distribution, calculated according to Burkardt, 2014 and Zaiontz, 2021)

The current study suggests that the use of paper-and-pencil data collection still results in marginally higher levels of accuracy and appears to replicate the findings of Tarbox et al. (2010). The question remains as to whether this marginal difference is of any clinical importance. It would be unwarranted to establish a criterion of clinical significance without consideration of the specific behaviors of interest and their baseline rates. For example, a .04 difference in accuracy may be clinically important for low-frequency aggressive behavior, whereas it might not be for high-frequency stereotypy. In order to provide additional context, Fig. 5 presents the distribution of inter-observer agreement (IOA) values (used here as an analogue for accuracy) obtained from a selection of the functional analysis literature as reported in Virues-Ortega (2022a). This ancillary analysis suggests that a range of .04 accounts for one standard deviation and a nontrivial one fifth of the range of usable values reported in this sample of the literature (0.8–1.0). Moreover, IOA values ranging .04 from one another have a cumulative probability of being found in the literature of up to 43%, again underlying that a .04 difference in accuracy may not be trivial. However, given the fact that all methods yielded accuracy scores greater than .80, where the general convention is to accept agreement/accuracy scores of .80 or greater (Cooper et al., 2019), the difference may be anecdotal for practical purposes.

The current findings result from an analysis of a dataset derived from the observational data collected from a diverse range of video sessions that include multiple target behavior topographies (self-injurious, destructive, aggressive behaviors) and a range in terms of the number of occurring events. It is surprising that the data also suggest that paper-and-pencil observation is still likely to be more accurate when the number of recordable events is relatively high (a proxy metric for behavior observation difficulty in this context). Future analyses should evaluate alternative indicators of behavior observation difficulty such as number of concurrent target behaviors and operational definition complexity. We controlled for participant observation experience and training, which are known confounds of observation performance (Mash & McElwee, 1974; Wildman et al., 1975). However, it is unclear whether our findings would be typical of more experienced observers, or whether behavior observation practice could effectively mediate the difficulty–accuracy relation. The potential impact of observation difficulty should be evaluated experimentally and not just as a post hoc correlational analysis, and such findings should be replicated with more varied and extensive samples of behavior.

One factor that may have influenced the apparent superiority of paper-and-pencil observation may have been the familiarity and simplicity of using a basic tool relative to the complexities involved in learning how to use a computer-based system. It should be noted that participants used the paper-and-pencil method during the behavior observation training protocol and received no performance-based training for the other two methods. We could start to address this concern by examining the aggregated session-by-session performance for each of the three methods over time. Specifically, Fig. 3 does not reveal an apparent ascending trend (which would be suggestive of a learning effect) for any of the three methods (see a trend-stationarity test in the Supplementary Online Material, Table B, for further details).

It is also possible that computer-aided observation required a larger number of in-session responses to complete key routines including recording an event, deleting an incorrectly recorded event, and replaying a specific video section. Engaging in longer chains of responses increases the potential for error (see Podofillini et al., 2013 for an empirical analysis of the linear relation between task complexity and operator error probability). In addition, increased effort may have influenced compliance with study procedures, particularly the intended use of video playback functions (see Hinz et al., 2014 for an applied demonstration of the relation between response effort and compliance in the context of behavior observation). Additional research may try and standardize the response effort and complexity of using any system in order to truly evaluate its utility and rule out such a confound.

Overall, without evidence that all computerized devices and the software systems, and their human-computer interfaces, are equivalent, which seems extraordinarily unlikely, the potential impact of the present work may be seen as restricted. In order to fully address this concern, it would have been necessary to conduct a detailed analysis of all existing systems or a component analysis to isolate the effect of common functionalities and interphase components. Either approach would have been impractical. Instead, we selected two considerably diverse systems such that, would convergent findings be obtained, a modest indication of generality would seem plausible.

Despite the apparent but marginal superiority of the paper-and-pencil method of collecting observational data suggested by this study (albeit that all methods yielded accuracy scores within accepted limits), there remain some ostensibly clear advantages to the use of computer-based systems that should be systematically evaluated, both in terms of practical application as well as outcome data. For example, the ability to automatically generate output files, produce descriptive summary statistics and graph the data may well offset the marginal loss in apparent accuracy. By contrast, the use of paper-and-pencil observation remains a well-established approach requiring minimal staff training and resources, which may be a critical advantage for low-resource communities.

As the complexity of computer-based systems for observational data collection increases, as seems inevitable with technological advances occurring exponentially and ubiquitously, there is a trade-off between the various features offered by a product and the basic ability to observe and collect observational data. Further research could evaluate these and other factors by, for example, controlling for complexity of use (e.g., the length of time and/or the number of key presses used for recording). In this context it is interesting to note that the overall observer accuracy for the two software systems utilized here (The Observer XT, Big Eye Observer) was almost identical in spite of the numerous differences in their respective user interfaces. Specifically, synced video functionality, number of discrete actions needed to complete key routines (e.g., score, delete, playback), and number of attention shifts required differed between the two observation systems.

Technological developments over the last few decades have provided scientists with a diverse set of observation tools with a concomitant impact on efficiency of use and accuracy of recording (Hall et al., 2014; Sarkar et al., 2006). Behavior observation capabilities have been improved by way of bespoke software for handheld and desktop devices (e.g., McKerchar & Abby, 2012; Virues-Ortega et al., 2011), sometimes incorporating the facility for video processing and retrospective coding (e.g., Hall et al., 2014). Studies using computer-aided observation often report high levels of interobserver agreement and/or accuracy, but direct comparisons are lacking. In addition, behavior-analytic studies rarely report the technology supporting the behavior observation process. Therefore, more systematic replications are needed to evaluate any favorable or deleterious effects that may be caused by computer-aided observation.

## Conclusions

The current analysis makes it possible to draw some tentative conclusions on the various methods utilized. First, our study suggests that it is possible to generate high accuracy of recording from newly trained observers with no previous experience in systematic observation using a range of observation methods with naturalistic observation materials. Second, paper-and-pencil observation induced a marginally superior level of accuracy relative to computer-aided observation systems. Third, there was a significant mediating effect of session *busyness* on observation accuracy for computer-aided observation systems but not for paper-and-pencil observation. Fourth, two computer-aided observation systems with very diverse user interface features induced near-identical observer accuracy. We believe that these findings help validate the continued use of both paper-and-pencil and computer-aided systems in behavior observation applications. They also highlight the need for additional research on key mediating factors including observer experience, observation difficulty, and the response effort involved in operating various systems.

## Supplementary Information

ESM 1 (DOCX 15 kb)
