# Supplementary material for: Accuracy of paper-and-pencil systematic observation versus computer-aided systems
Source: Behav Res Methods. 2022 Apr 27;55(2):855–66. doi: 10.3758/s13428-022-01861-0 (PMC10027644; doi:10.3758/s13428-022-01861-0)
Supplement: Supplementary file 1 — (DOCX 15 kb) [file 13428_2022_1861_MOESM1_ESM.docx]

*SUPPLEMENARY ONLINE MATERIAL*

Virues-Ortega et al.

**Table A**

*Common Attributes and Functionalities of Software-Based Observation Applications*

|  | The Observer XT | Big Eye Observer | Countee | Solomon  Coder |
| --- | --- | --- | --- | --- |
| Platform | PC | iOSX | Android, iOSX | PC |
| Behavior dimensions | Frequency, duration | Frequency, duration, partial interval, total interval | Frequency, duration | Frequency, durations, percentage, latencies, alternations |
| Main application | In video | In vivo | In vivo | In video |
| Clutter-free interphase | No | Yes | Yes | Yes |
| Video playback functions | Yes | No | No | Yes |
| In-session error correction | Yes | Yes | No | Yes |
| Session pausing | Yes | Yes | Yes | Yes |
| One-key/tap recording | Yes^1^ | Yes | Yes | Yes |
| One-screen recording | Yes | Yes | Yes | Yes |

*Note*. (1) Depending on the program configuration more than one keystroke may be needed to record an event.

**Table B**

*Presence of Time-Dependent Trends in in the Three Observation Methods*

|  | Dickey-Fuller Test | | | Trend Factor | | |
| --- | --- | --- | --- | --- | --- | --- |
|  | *Z(t)* | 5% Critical value | *p* value | Coefficient ±*SE* | *t* | *p* value |
| P&P | -3.26 | -3.60 | .072 | 0.001±0.001 | 0.56 | .584 |
| OXT | -2.76 | -3.60 | .211 | 0.004±0.003 | 1.55 | .144 |
| BEO | -3.43 | -3.60 | .005 | -0.002±0.001 | -1.22 | .243 |

*Notes.* All regression analyses assume an autoregressive factor of 1. BEO = Big Eye Observer, OXT = The Observer XT, P&P = paper-and-pencil; *SE* = Standard error.
